# Research on the Behavioral and ERP Characteristics Induced by the Availability Heuristic in Intuitive Decision-Making

**DOI:** 10.3390/s26010091

**Published:** 2025-12-23

**Authors:** Xilin Zhang, Wei Wang, Jue Qu, Sina Dang, Chao Wang

**Affiliations:** Air Defense and Antimissile School, Air Force Engineering University, Xi’an 710051, China

**Keywords:** availability heuristic, information visualization, ERPs, intuitive decision-making, experiment of behavior

## Abstract

Humans tend to rely on heuristic strategies for intuitive judgment during decision-making. Existing research proposes an availability heuristic, suggesting that individuals are inclined to use highly available information as a basis for judgment. To explore the behavioral and electrophysiological characteristics of the availability heuristic in information visualization, 24 right-handed participants were recruited for the experiment. Using behavioral and event-related potentials (ERPs) analysis techniques, within-subject behavioral and electroencephalogram (EEG) experiments were conducted under four conditions: polar coordinate system with higher number, polar coordinate system with lower number, Cartesian coordinate system with higher number, and Cartesian coordinate system with lower number. The behavioral results revealed that in the angle estimation task, the polar coordinate condition induced a more significant availability heuristic effect compared to the Cartesian coordinate condition, exhibiting smaller numerical estimation deviations. This indicates that the degree of semantic relevance between the available information and the target task is a critical factor determining the facilitative effect of such information on judgment. The ERPs results showed that the polar coordinate condition elicited smaller N2 and P2 amplitudes than the Cartesian coordinate condition during angle judgment, suggesting reduced semantic conflict and lower attentional demand in task processing under the polar coordinate condition. By providing behavioral and electrophysiological evidence of intuitive decision-making processes, this study lays a theoretical foundation for the rational application of intuitive effects in information visualization design. Furthermore, the findings imply that using available information semantically aligned with the target task can significantly enhance the effectiveness of the availability heuristic, thereby mitigating availability bias.

## 1. Introduction

Intuition and analysis represent two fundamental pathways in decision-making [1,2,3]. According to Dual-Process Theory (DPT) [4], intuitive decision-making is characterized by speed, automaticity, and a lack of deliberative effort. This is primarily because intuitive thinking often relies on an individual’s prior knowledge and is largely independent of conscious effort, thereby forming a cognitive simplification mechanism that significantly enhances decision-making efficiency [5]. Under time and information pressure, people tend to adopt heuristics as the primary mode of intuitive decision-making [6,7], including the Availability Heuristic, the Representativeness Heuristic, and the Anchoring and Adjustment Heuristic [8]. However, such cognitive simplification strategies are prone to triggering systematic decision-making biases [9]. Taking human–computer interface interaction as an example, when processing interface information, decision-makers’ judgments can be disproportionately influenced by easily retrievable instances or information from memory, significantly impacting decision outcomes and thereby undermining their rationality and accuracy [10]. In cognitive psychology, Tversky and Kahneman [11] defined this heuristic strategy of judgment based on the ease of information retrieval as the availability heuristic, categorizing it as a key component of intuitive thinking. Therefore, it is imperative to understand the behavioral characteristics and underlying electrophysiological evidence of this effect, and to explore effective methods for mitigating such biases.

Visual encoding, as the foundational basis of information visualization design, essentially involves the designer’s rational use of visual elements and encoding rules to support users in achieving more intuitive and efficient cognitive responses to information. The construction of visual encoding is grounded in human visual characteristics and cognitive psychological mechanisms, translating abstract information into concrete forms such as graphics, and conveying information through visual channels like size and color [12,13]. Munzner [14] identified two core components of visual encoding: geometric marks and visual channels. Taking numerical estimation tasks as an example, task information is often conveyed through geometric marks, with visual channels such as length, area, angle, and position enabling the visual mapping of quantitative information. Psychophysical research has demonstrated [15] that humans exhibit varying levels of accuracy in processing information from different visual channels, with spatial position perception showing particularly high correspondence to actual values [12]. The underlying reason for this phenomenon lies in the fact that, constrained by human perceptual characteristics, discrepancies can arise between visual perception and intuitive judgment during information processing. Studies have shown that in information visualization research, visual information serves as the core information carrier, and the quality of its semantic expression directly influences the efficiency of information transmission and the level of users’ cognitive load [16].

In the field of information visualization, research on the availability heuristic effect has evolved from early methodological development to the expansion of application scenarios and the optimization of mitigation strategies [17,18,19]. Early scholars primarily investigated the availability heuristic through interviews and textual analysis [20]. Chang later introduced the availability heuristic into data visualization scenarios, establishing a paradigm for exploring its effects across different domains [21]. Padilla et al. [22] further extended the research on the availability heuristic to visualization scenarios of visual information, providing methodological guidance for availability heuristic research related to visual graphs. Dimara et al. [23] examined the availability heuristic in visualization and proposed targeted strategies to mitigate its negative impacts. In the field of decision-making and cognitive control, Duma et al. [24] utilized high-density electroencephalography (hdEEG) technology, manipulated implicit temporal expectancy via the Dynamic Temporal Prediction (DTP) task to modulate proactive motor control, investigated the impact of implicit global temporal expectancy on the neurocomputational mechanisms of proactive motor control, and provided crucial references for exploring the heuristic decision-making processes and their neural substrates in time-constrained tasks. These studies provide an important theoretical foundation for understanding the mechanisms and modulation pathways of the availability heuristic effect. However, research on interventions addressing the availability heuristic in visual information perception remains in its early stages, lacking mechanistic explanations for the availability heuristic effect in visual information visualization and research on its regulatory methods. In this context, studying the biases induced by the availability heuristic through numerical formats is particularly relevant. This approach is motivated by the fact that numerical representations offer advantages over textual forms in terms of accuracy measurement, while graphical presentations facilitate rapid user information processing [25,26,27].

Contexts shape individuals’ decision-making patterns and individual differences in cognitive control by influencing the adaptive performance of cognitive control [28]. For the availability heuristic, the pre-configuration of heuristic information significantly influences the activation and application of the availability heuristic, with different types of heuristic cues leading to differentiated decision outcomes [9]. In visual interfaces, task-relevant interface information constitutes a critical factor influencing decision-makers’ judgments. Taking a target position judgment task in two-dimensional space as an example, coordinate system information often serves as a reference framework for assisting positional relationship judgments [29], and the specific selection of the reference system can substantially affect judgment results. Research in psychophysics further demonstrates that even when the physical attributes of target stimuli remain unchanged, variations in heuristic information may still induce systematic biases in individuals’ perception of targets [15]. To investigate the impact of the availability heuristic on individual decision-making, this study adopts a two-dimensional planar position perception judgment task as an exemplary scenario, employing polar and Cartesian coordinate systems as distinct types of heuristic information. By quantitatively analyzing the accuracy of users’ angular judgments of coordinate points under each coordinate system, we examine how coordinate systems influence the numerical estimation decision process, thereby elucidating the mechanisms through which heuristic information types affect individual decision-making.

As an important application of electroencephalogram (EEG) technology, event-related potentials (ERPs) record the brain’s electrical activity in response to specific stimuli or events, enabling the dynamic tracking of cognitive functions such as perception, attention, memory, and language comprehension [30]. Existing studies have confirmed that the characteristics of various ERP components exhibit systematic variations with changes in visual information, with N2 and P3 being among the components closely associated with visual information decision-making processes [31,32]. Research indicates that the N2 (also known as N200) component is a negative-going waveform associated with cognitive control and conflict monitoring, and is notably observed during stimulus discrimination and target judgment processes [33]. The N2 is sensitive to stimulus deviance, typically elicited between 180 ms and 350 ms after the presentation of a visual or auditory stimulus, and can be detected over central-parietal scalp regions [34]. During tasks involving target judgment, higher discriminability and judgability of information are associated with a shorter N2 latency and reduced amplitude, reflecting a more efficient cognitive process. The N2 is typically enhanced by deviations in the physical features or contextual congruity of a stimulus. Increased cognitive load often leads to greater negativity (i.e., a larger N2 amplitude) [35]. The P3 (or P300) component was first identified by Sutton et al. [36] in 1965. It is generally regarded as a positive component linked to the allocation of attentional resources and the processing of task difficulty, with a typical latency between 300 ms and 600 ms, most prominently recorded at central-parietal electrode sites. The P3 component reflects higher-order cognitive processing and aids in investigating mechanisms of attentional allocation and selection. Its occurrence usually signifies the recognition of a target stimulus. Greater allocation of attentional resources and higher cognitive load tend to result in an increased P3 amplitude or prolonged latency [37,38,39]. Therefore, ERP can serve as a physiological approach for analyzing individual decision-making processes.

This study focuses on a dual-dimensional exploration of the availability heuristic. First, using a numerical judgment task as the paradigm, it systematically compares the availability heuristic effects elicited by different types of available information, aiming to provide an in-depth analysis of the decision-making behavioral characteristics associated with this heuristic. Second, on the basis of verifying the existence of the availability heuristic, the research further investigates the impact of the type and numerical characteristics of available information on behavioral decision biases and neurophysiological features in decision-makers. By contrasting numerical estimation results with their corresponding differences in EEG activity, the study seeks to reveal the physiological correlates of the availability heuristic effect and explore potential intervention strategies for bias mitigation.

Based on existing research and theoretical foundations, this study proposes the following hypotheses:

**H1.**  *Drawing on relevant theoretical work regarding the availability heuristic [26,40,41], it is hypothesized that closer semantic proximity between the heuristic information and the target task will lead to a smaller deviation between the true value and the estimated value during individual decision-making. In this experiment, this would be reflected in a smaller numerical estimation bias under the polar coordinate condition compared to the Cartesian coordinate condition.*

**H2.**  *Based on previous studies of the availability heuristic [42], it is inferred that the numerical judgment of a target stimulus is significantly influenced by the heuristic information, demonstrating an intuition effect driven by the availability heuristic. Specifically, when the reference stimulus and the target stimulus form a high-number combination, the user’s estimated value of the target stimulus will be significantly higher than the true value; conversely, under a low-number combination, the estimated value will be significantly lower than the true value [43,44].*

**H3.**  *Integrating ERP research on visual information processing [30,45], it is proposed that the visually presented availability information will induce characteristic modulations in the typical ERP components N2 and P3. Changes in their amplitude and latency can serve as neurophysiological indicators for assessing the intensity of availability heuristic processing.*

## 2. Methods

### 2.1. Participants

Based on previous studies utilizing G*power [46,47], the sample size was determined a priori using G*power3.1. The calculation indicated that a minimum sample size of 22 participants was required, assuming a medium effect size (f = 0.25), an error probability (α) of 0.05, and a statistical power (1 − β) of 0.8 [48].

Accordingly, a total of 24 right-handed participants were recruited for the experiment. The mean age of the participants was 24.16 years (SD = 2.29). All participants had normal or corrected-to-normal vision and reported no history of neurological or psychiatric disorders. Two participants were excluded from the final analysis due to excessive EEG artifacts. Consequently, experimental data from 22 participants were included in the analysis. This study was conducted in accordance with the Declaration of Helsinki, and the protocol was approved by the Ethics Committee of Air Force Engineering University (Approval No.: 2025-AEC-15).

### 2.2. Stimuli

**Figure 1:**
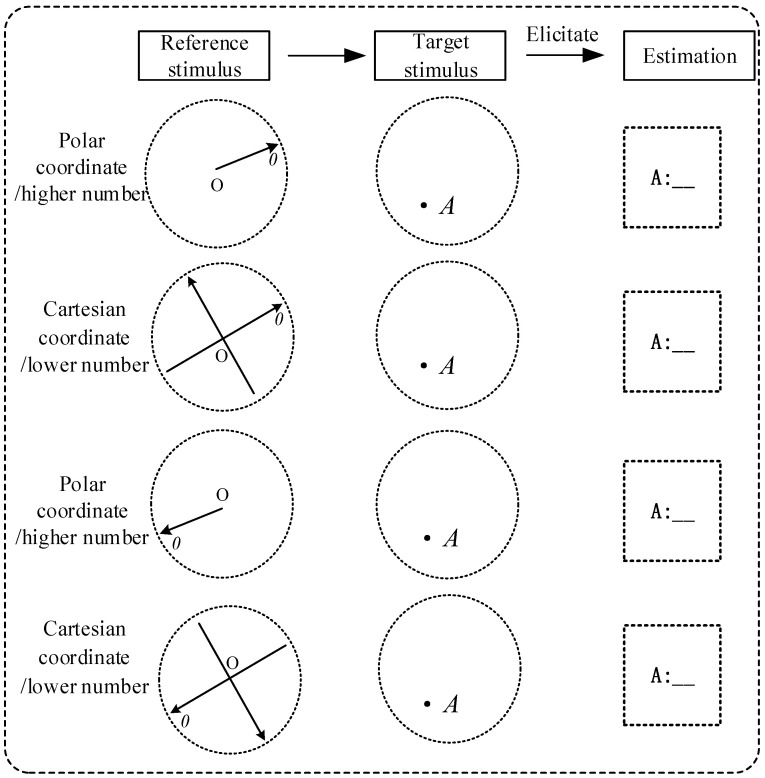
Sample stimulus in four conditions.

In this experiment, 4 monochromatic coordinate systems were used as reference stimuli, and 2 coordinate points served as target stimuli. Participants were required to judge the angle between the target coordinate point and the 0° axis (i.e., the target value) based on the reference coordinate systems, with the angle calculated in the counterclockwise direction. Angle greater than 180° was defined as higher number, and those less than 180° as lower number, with a fixed difference of 180° between higher and lower number. The target coordinate points were randomly generated within a circular area, and the positions of the reference coordinate systems were also randomly set; cases where the target points were located on the horizontal or vertical axes were excluded to avoid interference from special angles. Different reference coordinate systems corresponded to different heuristic information, while the target stimuli were coordinate points at fixed positions. The participants’ decision task was to estimate the specific angle value of the target coordinate points. A schematic diagram of the sample stimuli is shown in Figure 1.

A pre-test was conducted with ten participants who were required to perform numerical estimations of the sample stimuli within 1500 ms to validate the effectiveness of the stimulus materials. The results revealed a significant discrepancy between the estimated numbers and the actual numbers, which indicated that participants were unable to ascertain the true numbers of the targets.

### 2.3. Task

Building upon the experimental paradigm of the availability heuristic [11], the current study adapted the protocol by incorporating a numerical estimation task. Within each trial, participants were required to rapidly and accurately estimate the position of a target stimulus within a specified time limit. Participants first viewed the visual information presented in the initial image without providing any response. Following which, they were asked to evaluate the second image and provide a numerical estimation based on the information acquired from the first image. All instructions were carefully designed to avoid any terminology related to the availability heuristic or other concepts that might inadvertently influence participants’ decision-making. To ensure familiarity with the task procedure, a practice session consisting of 10 trials was administered prior to the formal experiment.

### 2.4. Equipment and Environment

Behavior experiment equipment: The experiment utilized E-Prime 2.0 software for stimulus presentation while simultaneously recording participants’ decision outcomes and reaction times.

ERP experiment equipment: EEG data were recorded using a Neuroscan NuAmps amplifier (Compumedics Limited, Sydney, Australia) with 32 Ag/AgCl electrodes mounted in an elastic cap according to the international 10–20 system. The ground electrode was positioned at the midpoint between Fp1 and Fp2, and the reference electrode was placed at Cz during recording. Offline analysis included re-referencing to the average of the TP7 and TP8 electrodes located on bilateral mastoids. The data were filtered with a bandpass of 0.5–80 Hz and sampled at a rate of 500 Hz per channel. All electrode impedances were maintained below 5 kΩ. Data acquisition and processing were performed using Curry 7 software. Epochs were extracted from 200 ms prior to 1000 ms following the onset of the target stimulus, with the 200 ms pre-stimulus interval serving as the baseline. Trials containing ocular artifacts or other artifacts exceeding ±100 μV were excluded from analysis.

**Figure 2:**
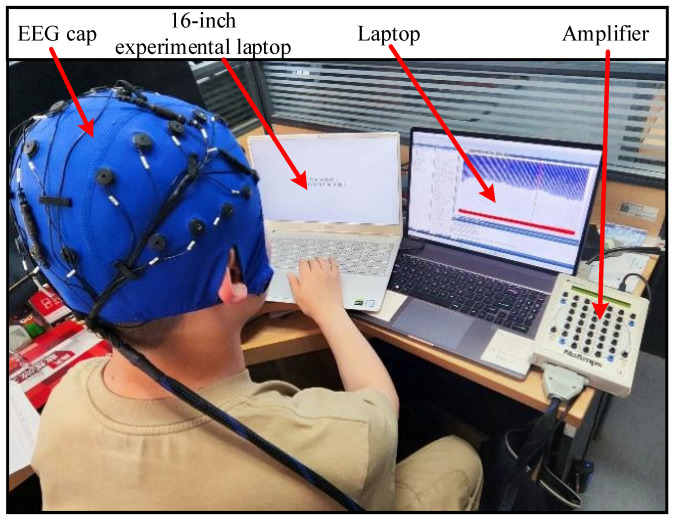
ERP experimental system.

Experimental environment: The experiment was conducted in a dedicated ERP/behavioral laboratory equipped with sound-attenuating, magnetically shielded, and adjustable lighting conditions. Visual stimuli were presented on a 16-inch LCD monitor, and participants provided responses via a keyboard according to task requirements. During the formal experiment, each participant was seated in a comfortable chair in a quiet, normally lit environment. They were instructed to fixate on the center of the computer screen positioned approximately 75 cm away, and to complete multiple trials of the experimental task. The ERP experimental system is shown in Figure 2.

### 2.5. Experimental Design and Procedures

**Figure 3:**
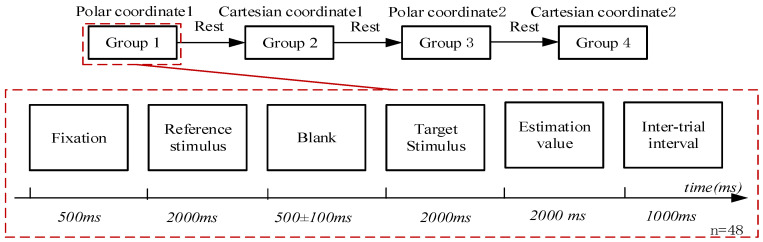
Experimental procedure.

The experiment employed a 2 × 2 within-subjects design, comprising four experimental conditions: Condition 1 (“Polar Coordinate with higher number”), Condition 2 (“Cartesian Coordinate with higher number”), Condition 3 (“Polar Coordinate with lower number”), and Condition 4 (“Cartesian Coordinate with lower number”). To accommodate the four conditions and meet the signal-averaging requirements of ERP analysis, the experiment was organized into four blocks: Polar Coordinate block 1, Cartesian Coordinate block 1, Polar Coordinate block 2, and Cartesian Coordinate block 2. Within each block, trials with higher and lower numbers were presented randomly, totaling 48 trials per block and 192 trials in entirety. The experimental procedure is illustrated in Figure 3.

In each trial, a central fixation cross was first displayed for 500 ms, followed by the heuristic stimulus image. A blank screen then appeared for a variable interval of 400–600 ms, after which the target stimulus was presented. Finally, a decision interface was displayed, prompting participants to provide a numerical estimate for the target. Each trial was separated by an inter-trial interval of 1000 ms, and the entire experiment lasted approximately 30 min.

### 2.6. Statistical Analysis

#### 2.6.1. Behavioral Data

For each participant, the mean estimated value under each of the four conditions was calculated. Deviation scores were derived by subtracting the mean correct value from the mean estimated value (i.e., Deviation = Mean Estimate–Mean Correct Value). A repeated-measures analysis of variance (ANOVA) was conducted to analyze the estimates across the four conditions, with the alpha level set at 0.05.

#### 2.6.2. ERP Data Analysis

**Figure 4:**
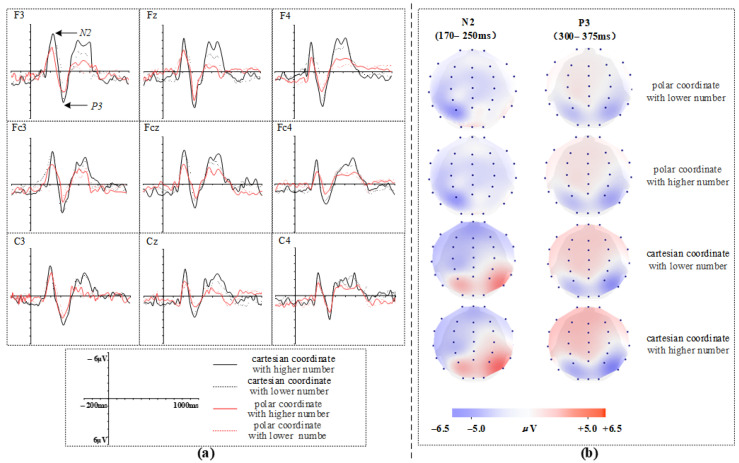
(**a**) Grand average ERPs waveforms elicited by different conditions; (**b**) Topographic maps for different conditions.

In the present experiment, participants were required to sequentially complete information processing stages including the perception of coordinate system information, numerical judgment of target points, and estimation of specific angles. Consistent with the conclusions of the literature review presented earlier, different types of coordinate systems in the experimental context induce varying degrees of information perception conflict and differentiated attention resource allocation. These differences are reflected in the temporal characteristics of ERP components across distinct scalp regions. In line with previous research [31,32], and based on the grand-average waveforms and topographic maps illustrated in Figure 4, the mean amplitudes of the N2 (180–250 ms) and P3 (300–375 ms) components were selected for analysis. The ERP’s amplitude was calculated within the respective time windows corresponding to each component.

Electrode selection focused on nine sites spanning frontal and central regions: the frontal group (Fz, F3, F4), frontal-central group (FCz, FC3, FC4), and central group (Cz, C3, C4). Data were analyzed using a repeated-measures analysis of variance (ANOVA) with four within-subject factors: coordinate type (polar, Cartesian), numerical value (high, low), laterality (left, midline, right), and brain region (frontal, frontal-central, central). The alpha level was set at 0.05 for all statistical tests. The Bonferroni method was applied to correct *p*-values for multiple comparisons, and the Greenhouse-Geisser correction was used to adjust degrees of freedom in cases where the assumption of sphericity was violated.

## 3. Results

### 3.1. Behavioral Results

**Figure 5:**
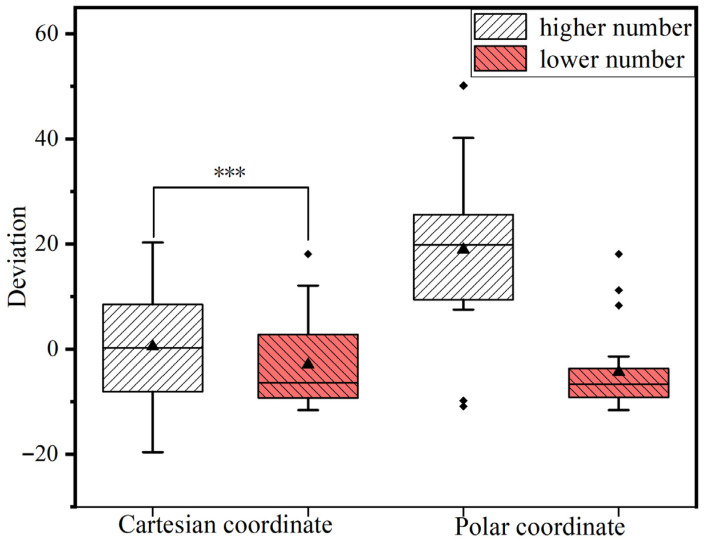
The deviation of the estimate values from the correct answer in four conditions. *** represents statistically significant at 0.001 level (*p* value < 0.001).

A repeated-measures analysis of variance (ANOVA) was conducted on participants’ estimation values, with the results illustrated in Figure 5. The within-subjects effects analysis revealed a significant interaction between heuristic information type (coordinate system) and numerical value type (F = (1, 21) = 25.83, *p* < 0.001, $\eta_{P}^{2}$ = 0.552). To decompose this interaction, simple effects analyses were performed. The results indicated that under the Cartesian coordinate condition, there was a significant difference between the estimates for higher and lower numbers (F = (1, 21) = 2.191, *p* < 0.001, $\eta_{P}^{2}$ = 0.431), overestimated higher numbers and underestimated lower numbers. In contrast, under the polar coordinate condition, no significant difference was observed between the estimates for higher and lower numbers (F = (1, 21) = 26.04, *p* = 0.154 > 0.1, $\eta_{P}^{2}$ = 0.094). These behavioral results demonstrate a significant availability heuristic effect when participants made angle estimations based on the two coordinate systems, with the magnitude of the induced bias differing substantially between the systems.

### 3.2. ERP Results

#### 3.2.1. N2 (180–250 ms)

**Figure 6:**
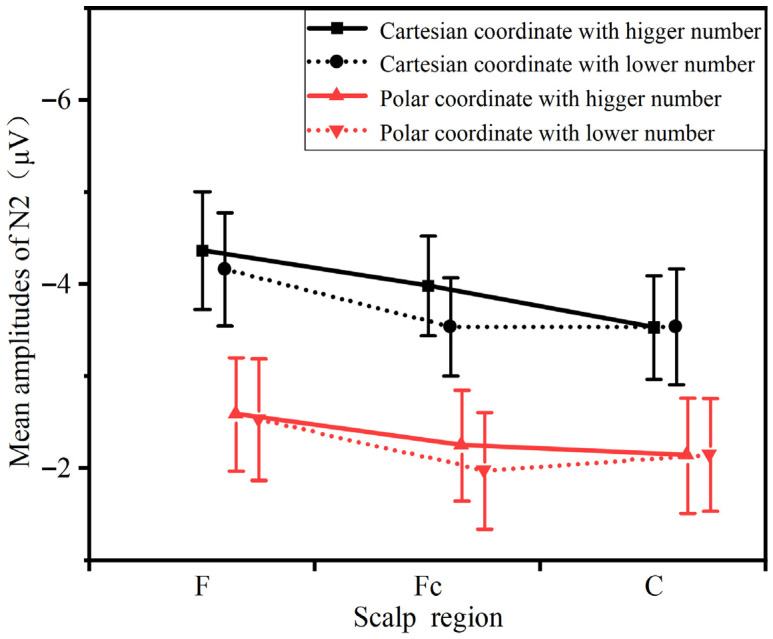
The comparison of the mean amplitudes of N2 between different conditions and scalp regions.

A repeated-measures analysis of variance (ANOVA) was conducted on the mean N2 amplitudes, with results presented in Figure 6. The analysis revealed a marginally significant interaction between heuristic information type and electrode site (F = (2, 42) = 13.315, 0.05 < *p* = 0.07 < 0.1, $\eta_{P}^{2}$ = 0.99). Additionally, a significant main effect of information type was observed (F = (1, 21) = 10.956, *p* = 0.022 < 0.05, $\eta_{P}^{2}$ = 0.999), indicating that the Cartesian coordinate condition elicited larger N2 amplitudes than the polar coordinate condition. To further examine the interaction between electrode site and information type, simple effects analyses were performed. The results showed significant differences between information types in the frontal region (F = (1, 21) = 23.027, *p* = 0.027 < 0.05, $\eta_{P}^{2}$ = 0.998) frontal-central region (F = (1, 21) = 19.151, *p* = 0.033 < 0.05, = 0.997), and central region (F = (1, 21) = 13.9, *p* < 0.001, $\eta_{P}^{2}$ = 1.01). Furthermore, the topographic maps revealed no significant main effect of numerical value type.

#### 3.2.2. P3 (300–375 ms)

**Figure 7:**
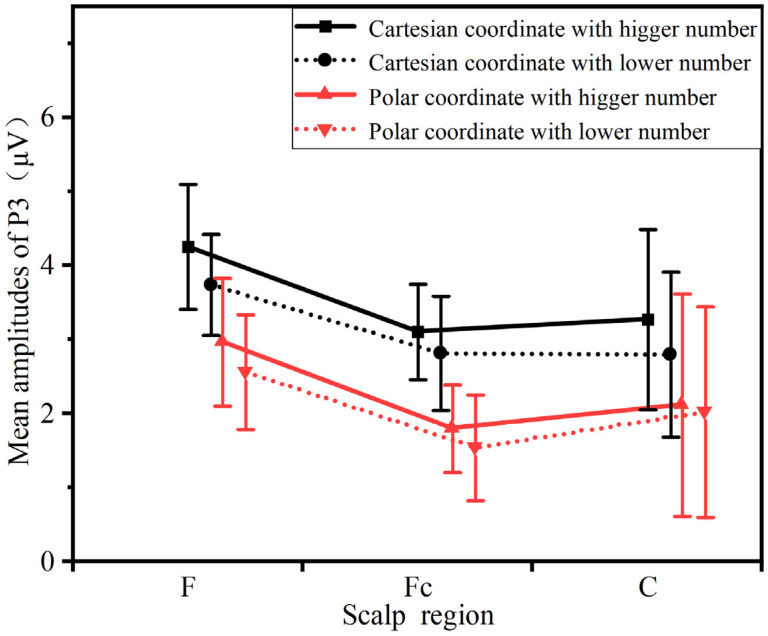
The comparison of the mean amplitudes of P3 between different conditions and scalp regions.

A repeated-measures ANOVA was performed on the mean amplitude of the P3 component, with the results displayed in Figure 7. The analysis revealed a significant main effect of information type (F = (1, 21) = 8.312, *p* = 0.048 < 0.05, $\eta_{P}^{2}$ = 0.994). A marginally significant interaction was observed between information type and electrode site (F = (2, 42) = 10.656, 0.05 < *p* = 0.086 < 0.1, $\eta_{P}^{2}$ = 0.914), and a significant interaction emerged between information type and laterality (F = (2, 42) = 5.009, *p* = 0.041 < 0.05, $\eta_{P}^{2}$ = 0.268). To further decompose the interaction between electrode site and information type, simple effects analyses were conducted. These analyses indicated that the P3 amplitude was significantly larger under the Cartesian coordinate condition compared to the polar coordinate condition in the frontal region (F = (1, 21) = 25.301, *p* = 0.011 < 0.05, $\eta_{P}^{2}$ = 0.535) and the central region (F = (1, 21) = 2.387, *p* = 0.003 < 0.05, $\eta_{P}^{2}$ = 0.263). However, the effect was not significant in the frontal-central region (F = (1, 21) = 8.029, *p* = 0.225 > 0.1, $\eta_{P}^{2}$ = 0.586). Furthermore, the topographic maps revealed no significant main effect of numerical value type.

## 4. Discussion

The availability heuristic, as a classic topic in psychology and neuroscience, has accumulated a substantial body of theoretical and empirical research. However, establishing its systematic theoretical connection with human intuitive thinking, and further elucidating their respective mechanisms and manifestations in information visualization contexts, remain critical challenges for contemporary academia. This study investigated the availability heuristic effect of different coordinate systems in a two-dimensional position perception and numerical estimation task. By designing a numerical estimation task combined with ERP experiments, the study yielded the following key findings: First, it proposed a novel paradigm for studying the availability heuristic effect and its associated biases using numerical estimation tasks under different coordinate systems, providing an extensible experimental framework for the field. Second, it demonstrated that the temporal dynamics and scalp distribution of EEG activity can effectively characterize the dynamic process of visual information processing, offering direct neural evidence for the analysis of intuitive decision-making. By integrating interdisciplinary perspectives from information visualization design and cognitive psychology, this study significantly enhances the validity of intuitive bias detection in visual interactions and provides a methodological framework with both theoretical depth and practical value for deepening the understanding of the bidirectional relationship between users’ cognitive characteristics and visual encoding elements.

### 4.1. Behavioral Analysis

The behavioral results align with Hypothesis H1: in the angle judgment task, the numerical estimation bias induced under the polar coordinate condition was significantly smaller than that under the Cartesian coordinate condition. Given that the angle judgment of coordinate points served as the target task across all four experimental conditions, it can be inferred that the degree of relevance between the reference information and the target task is a critical factor underlying the observed behavioral differences. According to the experimental design, this relevance is primarily reflected in the differences in visual representation of angular semantics [49]. The polar coordinate system is inherently used to define the angle and distance of a target, whereas the Cartesian coordinate system defines the directed distances of a target. Consequently, in angle judgment tasks, the polar coordinate system demonstrates higher priority and selectivity, yielding a more pronounced availability heuristic effect. This finding suggests that the heuristic association between the coordinate system and the coordinate point, rather than the specific form of the coordinate system itself, is more likely to trigger the availability heuristic. The underlying mechanism lies in the fact that the cognitive transformation complexity between the decision goal and the available information directly influences the heuristic effect: a simpler transformation process leads to a stronger heuristic effect and correspondingly higher decision accuracy.

Furthermore, the behavioral results revealed that under the Cartesian coordinate condition, participants significantly overestimated high values and underestimated low values, whereas under the polar coordinate condition, no significant bias was observed for either high or low values, thus validating Hypothesis H2. This phenomenon can be attributed to the structural differences between the coordinate systems: the polar system involves only a single direction (with counterclockwise defined as the positive angular direction), while the Cartesian system incorporates both horizontal and vertical axes. The resulting difference in cognitive load during rapid angle judgment likely accounts for the variation in bias significance.

These conclusions are consistent with existing human factors research [20,26,50], which posits that closer semantic proximity between reference information and the target task reduces the cognitive effort required, minimizes systematic biases, and enhances the availability heuristic effect. Moreover, this study further indicates that a feasible strategy for mitigating the negative impact of availability bias is to provide semantically relevant heuristic information prior to presenting the target information, thereby guiding users to employ the availability heuristic more efficiently. By presenting auxiliary information that is semantically closely aligned with the target task, the availability heuristic can be effectively activated, significantly enhancing the fluency and accuracy of decision-making.

### 4.2. Analysis of ERP Components

In the present experiment, although no explicit instructions were given regarding the evaluation of visual stimuli, the ERP results demonstrated that the Cartesian coordinate condition elicited larger N2 and P3 amplitudes over the frontal, central, and frontal-central regions, which is consistent with Hypothesis H3. During the experiment, participants completed a series of information processing tasks, ranging from the perception of coordinate system information and numerical judgment of target points to angle estimation. In this process, the perception of information characteristics of the coordinate system triggered intuitive processing dominated by the availability heuristic; the conflict detection between these information characteristics and the target task was reflected in the temporal dynamics and amplitude characteristics of the N2 component. Subsequently, participants were required to make angle judgments on coordinate points based on the information characteristics of the coordinate system. Different angle judgment rules led to differences in the amount of attentional resources occupied during the judgment process, and this difference was ultimately manifested in the temporal dynamics and amplitude characteristics of the P3 component.

Analysis of the N2 component revealed significantly smaller amplitudes under the polar coordinate condition compared to the Cartesian condition. The N2 component is generally regarded as reflecting stimulus discrimination, target evaluation, and is implicated in cognitive control and conflict monitoring [27]. Furthermore, it plays a significant role in studies of attention, memory, and decision-making processes. Therefore, the observed N2 differences in this study suggest that different types of heuristic information elicited varying degrees of N2 responses during target judgment. In the present task, participants were required to interpret coordinate information and perform an angle judgment regarding target position. The polar coordinate system directly provides angular information, involves lower conflict processing and computational demand, and renders angle judgment more akin to a process of positional recognition. This likely reduced the allocation of attentional resources and the overall decision difficulty, leading to the observed attenuation in N2 amplitude. In contrast, the Cartesian coordinate system, with its horizontal and vertical axes, entails more directional information and introduces a degree of semantic conflict with the angle judgment process, thereby demanding greater attentional resources and increasing decision difficulty, which was reflected in the enhanced N2 amplitude.

Analysis of the P3 component indicated significantly smaller amplitudes in the polar coordinate condition relative to the Cartesian condition. The P3 component is closely associated with the allocation of attentional resources to task-relevant stimuli [30]. In the angle judgment task used here, the polar coordinate system provided more direct angular information, facilitating participants’ judgments and reducing the demand for attentional resources, as evidenced by the smaller P3 amplitude. Moreover, P3 is highly sensitive to task difficulty. Compared to the Cartesian system, the polar system entails lower informational complexity in terms of quadrants and directional cues, resulting in lower cognitive load for angle judgment and less attentional investment during decision-making, which further accounts for the reduced P3 amplitude.

### 4.3. Analysis of Application Prospects

This study adopted a visual reference-based angle estimation task paradigm, derived from the core cognitive-behavioral pathway of “visual information presentation, intuitive decision-making and action execution” in real-world scenarios. Its design logic aligns closely with the interaction requirements of complex information interfaces such as radar search interfaces and dashboards. By exploring of basic angle estimation issues, this study lays a theoretical foundation for the subsequent extension to the intuitive design of complex information radar interfaces. Specifically, the conclusions from the comparative analyses of polar coordinates and Cartesian coordinates in this paper can be directly extended to the intuitive design practice of radar search interfaces, providing a targeted approach for constructing design solutions that assist users in making efficient intuitive decisions. Preliminary design validation results indicate that using this study’s conclusions as the design basis for radar search interfaces can significantly enhance the efficiency and accuracy of users’ angle estimation decision-making. However, the design of complex interfaces needs to integrate multi-dimensional factors, and relying solely on the basic conclusions of this study is insufficient to support a complete design system. Future research should further deeply integrate the findings of this study with interface design methodologies, realize the transformation of theoretical discoveries into practical applications through interdisciplinary integration, and promote the systematic development of intuitive interaction design for complex information interfaces.

### 4.4. Implications and Limitations

#### 4.4.1. Theoretical and Practical Implications

Focusing on the availability heuristic as a representative strategy of intuitive decision-making, this study systematically investigated the intuitive effects within information visualization, providing an in-depth analysis of the behavioral characteristics and electrophysiological evidence of intuitive decisions in data visualization scenarios. By examining the availability heuristic effect in visual encoding and information processing, it elucidated the relationship between the properties of available information and the ERPs components N2 and P3, confirming these components as potential neural markers for quantifying availability bias. The findings demonstrate that ERP technology effectively establishes a crucial link between objective information design and human subjective behavior and cognitive processes, offering a scientific basis for uncovering the neural mechanisms of human–computer interaction. This implies that leveraging neuro-design tools such as ERP has the potential to provide visualization designers with novel quantitative perspectives for evaluating interaction performance.

The theoretical and practical contributions of this study are primarily reflected in its use of information visualization to reveal manifestations of the availability heuristic, thereby offering clear directions for mitigating availability bias and optimizing visualization design. Taking the interface design of weapon command and control systems as an example, although intuitive decision-making strategies such as the availability heuristic can improve decision efficiency, the resulting biases may impair user judgment. Based on our findings, designers can anticipate potential user biases from a visualization perspective and strategically adjust visual encoding strategies, thereby significantly enhancing the accuracy and timeliness of user decisions. This provides scientifically grounded guidance for optimizing the design of complex human–computer interaction systems.

#### 4.4.2. Limitations and Future Work

This study has certain limitations. Firstly, as individuals’ subjective and intuitive thinking patterns, the availability heuristics may exhibit certain differences in their effects across different individuals, a phenomenon closely associated with variations in individuals’ cognitive styles, knowledge backgrounds, and cognitive abilities. Given the limited sample size of this study and the inherent complexity of measuring individual difference variables, the relevant influence mechanisms have not been systematically analyzed. Future research may adopt standardized measurement tools such as cognitive style inventories and specialized mathematical ability tests to further explore the moderating effects of cognitive styles and mathematical abilities on user performance and subjective evaluations. Nevertheless, the implications of this work for universal design principles related to the availability heuristic remain valid. Second, the use of a numerical estimation task in this study was intended to facilitate the quantification of availability bias, yet it may not fully accommodate other complex factors. Other types of experimental tasks could be more suitable for such investigations and should be explored in future research. Finally, in visualization design, visual elements possess multi-dimensional attributes that must be considered holistically rather than from a single perspective. Future studies should further examine these multi-dimensional factors to enhance the robustness and generalizability of design principles.

## 5. Conclusions

This study investigates the influence of the availability heuristic on intuitive decision-making in information visualization, while exploring the neural correlates between visual encoding and the availability heuristic effect from a neurodesign perspective. The findings demonstrate that the polar coordinate system elicits a stronger availability heuristic effect compared to the Cartesian coordinate system in angle estimation tasks, highlighting the differential facilitative effects of information types on target tasks. The ERP components N2 and P3 served as neural markers of the availability heuristic effect. Specifically, information presented in the polar coordinate system showed higher processing efficiency during the early stage of stimulus detection (reflected by reduced N2 amplitude) and required less attentional resource allocation (indicated by decreased P3 amplitude). Based on these findings, this study suggests that aligning the characteristics of target information with appropriate visual representations can effectively enhance the usability of visualization designs. By providing an innovative perspective on the intuitive decision-making process centered on the availability heuristic in information visualization and its underlying neural mechanisms, this research establishes a theoretical foundation for interdisciplinary integration of cognitive neuroscience and visualization design.

## Data Availability

Data will be made available on request.
